# A Pilot Randomised Control Trial Exploring the Feasibility and Acceptability of Delivering a Personalised Modular Psychological Intervention for Anxiety Experienced by Autistic Adults: Personalised Anxiety Treatment-Autism (PAT-A)

**DOI:** 10.1007/s10803-023-06112-5

**Published:** 2023-09-20

**Authors:** Jacqui Rodgers, Samuel Brice, Patrick Welsh, Barry Ingham, Colin Wilson, Gemma Evans, Katie Steele, Emily Cropper, Ann Le Couteur, Mark Freeston, Jeremy R. Parr

**Affiliations:** 1 Population Health Sciences Institute, Newcastle University, Newcastle Upon Tyne, UK; 2 Cumbria, Northumberland, Tyne and Wear NHS Foundation Trust, Newcastle Upon Tyne, UK; 3 Newcastle Upon Tyne Hospitals NHS Foundation Trust, Newcastle Upon Tyne, UK; 4 School of Psychology, Newcastle University, Newcastle Upon Tyne, UK

**Keywords:** Autism, Anxiety, CBT, Modified, Randomised, Personalised treatments

## Abstract

Anxiety is commonly experienced by autistic people and impacts on quality of life and social participation. New anxiety interventions are required to effectively meet the needs of autistic people. Personalised Anxiety Treatment-Autism (PAT-A©) is a bespoke, modular approach to treating anxiety in up to 12 sessions. This study explored the feasibility and acceptability of delivering PAT-A© in the UK National Health Service (NHS). A single-blind randomised controlled trial design. Thirty-four autistic adults were recruited via clinical services and randomised to receive either PAT-A© or enhanced treatment as usual (CCSP). Outcome assessments relating to anxiety, quality of life and related constructs were completed at baseline, immediately post intervention; and at 3 and 12 months. Seventy-one percent of the PAT-A© group and 65% of the CCSP met diagnostic threshold for at least three anxiety disorders. Retention was good across both groups, with 82% (N = 14/17) completing the full course of PAT-A© and 71% (N = 12/17) attending both psychoeducational sessions in CCSP. 94% in PAT-A© and 82% in CCSP completed some follow up assessment 3 months post-intervention. Thematic analysis of interview data revealed that many participants valued the personalised approach, developed transferable skills and experienced positive changes to their anxiety. Participants were willing to be recruited and randomised, PAT-A© was feasible to deliver in the NHS and the trial methods and materials were acceptable. Our findings indicate that a fully powered clinical and cost-effectiveness trial of PAT-A© is warranted.

**Supplementary Information:**

The online version contains supplementary material available at 10.1007/s10803-023-06112-5.

Anxiety is common for autistic people, with a prevalence rate of 42% for autistic adults (Hollocks et al., 2019). Anxiety limits quality of life (Mason et al., 2019) and impacts on employment and education (Harmuth et al., 2018). Since anxiety may interact with autistic traits (Moore et al., 2021; South & Rodgers, 2017), autistic people may experience anxiety differently (Spain et al., 2017). Autistic people may experience alexithymia (difficulties identifying and describing emotions) (Kinnaird et al., 2019) and have difficulties coping with distress or regulating emotions (Weiss et al., 2014). Identifying these difficulties may guide delivery of more effective psychological therapy (National Autistic Society (NAS), 2021).

Developing effective anxiety interventions for autistic people is a key priority in the United Kingdom (Department of Health, 2014; James Lind Alliance, 2016; National Institute for Health and Care Excellence (NICE), 2012). Cognitive Behavioural Therapy (CBT) is typically the recommended first line treatment for anxiety (NICE, 2011). CBT and mental healthcare require adjustments and adaptations for autistic people (NAS, 2021; NICE, 2012), including for example: a therapist who understands autism; use of technology; use of written and visual material; a more concrete, structured approach; and involving supporters. Clinicians report that ‘autism specific’ adaptations accounted for around 40% of the ‘active ingredients’ that made CBT with autistic people more effective (Spain & Happé, 2020).

There is limited, but promising, evidence for the effectiveness of adapted CBT for autistic adults experiencing anxiety (Spain et al., 2015). A meta-analysis reported CBT to be effective in treating affective disorders (primarily anxiety) experienced by autistic people; however, effect sizes were small to medium (Weston et al., 2016). Adapted CBT has also demonstrated promise in treating phobia experienced by autistic adults (Maskey et al., ). A recent systematic review reported inconsistencies regarding the effectiveness of CBT with autistic adults but proposed that mindfulness-based therapies may be an effective anxiety treatment (Menezes et al., 2022). Additionally, a review of studies reporting on successful CBT interventions with autistic young people concluded such approaches offer more adjustments than those recommended in NICE guidelines (Walters et al., 2016). One important consideration yet to be addressed in the development of psychological therapies for anxiety, is that autistic people are more likely to experience multiple anxiety disorders concurrently (Joshi et al., 2013). Interventions that target one specific anxiety disorder (or anxiety in one specific situation) may thus address only part of an autistic individual’s anxiety experience. Autistic people may therefore benefit from modular approaches which can be used flexibly to offer a personalised intervention, potentially able to address anxiety across multiple contexts.

Anxiety in social situations (Spain et al., 2018) and phobias/situation specific anxiety (Lever & Geurts, 2016) are particularly common for autistic people and there is evidence supporting adapted CBT as an effective treatment for both (Maskey et al., 2019a; Maskey et al., 2019a, 2019b, 2019c, 2019d; Spain et al., 2015). Maskey et al., (2019a, 2019b, 2019c, 2019d) found acceptability and feasibility of using CBT in a virtual reality setting. Research also identified Intolerance of Uncertainty (IU) (Carleton, 2016) as a transdiagnostic factor in anxiety experienced by autistic people (Jenkinson et al., 2020). The Coping with Uncertainty in Everyday Situations (CUES) (Rodgers et al., 2018) psychological therapy was developed for autistic people to address IU and has demonstrated acceptability, feasibility and promising effectiveness. Behavioural interventions, such as mindfulness-based stress reduction, have also demonstrated efficacy in alleviating anxiety symptoms for autistic adults (Gaigg et al., 2020). Finally, the importance of supporting autistic people to develop the requisite skills in understanding and describing emotions has been persistently highlighted as an important modification to improve the efficacy of CBT as a transdiagnostic anxiety treatment for autistic people (Walters et al., 2016).

In this context, we developed the Personalised Anxiety Treatment-Autism (PAT-A), which has been designed as a modular intervention for autistic people. PAT-A© aims to respond to the individual’s anxiety presentation(s) by offering treatment modules for two common anxiety disorders (social anxiety, and situational anxiety/phobia) as well as three modules based on transdiagnostic constructs that appear related to anxiety (IU, emotional literacy, and distress tolerance). The modular nature of PAT-A© means that it can be applied flexibly to respond to the adjustments needed to health care delivery and to an individual’s anxiety presentation(s), which might include multiple anxiety disorders and/or across contexts.

The primary purpose of this trial was to explore the feasibility and acceptability of PAT-A© by recruiting autistic people who experience a range of anxiety disorders, through UK National Health Service (NHS) services. The feasibility and acceptability aims relate to the design and delivery of (i) the PAT-A© intervention including the training of therapists; the retention and engagement of participants; and assessment and formulation procedures leading to treatment planning and (ii) aspects of the randomised controlled trail such as recruitment, and randomisation procedures; and selection, timing, and completion of outcome measures. Other outcomes, such as the potential reduction in anxiety and increased engagement in ‘target situations’ were also included to inform future trial design and clinical use.

## Methods

### Design

Study methods are reported in detail in the published protocol (Parr et al., 2020) and summarised here. The PAT-A© pilot feasibility and acceptability randomised controlled trial (pilot RCT) included blind-to-group assessment. Participants were randomly allocated to either an intervention group (Personalised Anxiety Treatment-Autism; PAT-A©) or control group (Current Clinical Services Plus; CCSP). A favourable ethical opinion was provided (Wales Research Ethics Committee 5 (18/WA/0014); NHS Health Research Authority (IRAS ID: 235805). The trial was registered during recruitment (ISRCTN15881562).

### Participants

Thirty-four autistic adults were recruited via NHS clinical teams (adult mental health and adult autism diagnostic services) within Cumbria, Northumberland, Tyne and Wear NHS Foundation Trust. The demographic characteristics of participants are reported in Table 1.

**Table 1:** Demographic characteristics of the sample

	PAT-A© (N = 17)	CCSP (N = 17)
**Gender N (%)**		
Male	11 (64.7)	11 (64.7)
Female	6 (35.3)	5 (29.4)
Non-binary gender	0 (0.0)	1 (5.9)
**Age mean (SD) [range]**	39.5 (15.3) [21–62]	33.1 (10.2) [18–56]
**Ethnicity N (%)**		
White British	16 (94.1)	16 (94.1)
Mixed race	1 (5.9)	1 (5.9)
**Employment N (%)**		
Employed full time	2 (11.8)	2 (11.8)
Employed part time	2 (11.8)	1 (5.9)
Full time student	2 (11.8)	4 (23.5)
Volunteer	2 (11.8)	3 (17.6)
Unemployed	8 (47.1)	7 (41.2)
Retired	1 (5.9)	0 (0.0)
**Marital status N (%)**		
Single	13	12
Married	3	4 (23.5)
Cohabiting	1 (5.9)	1 (5.9)
**Age at autism diagnosis mean (SD) [range]**	37.1 (15.2) [11–61]	30.4 (9.8) [11–49]
**SRS-2 score (α = .94)**		
Missing N (%)	2 (11.8)	2 (11.8)
Mean raw score (SD)	125.1 (22.1)	110.1 (30.0)
**Toronto alexithymia scale mean (SD)**		
Difficulty describing feelings	19.5 (3.1)	19.2 (2.9)
Difficulty identifying feelings	26.0 (5.0)	23.8 (5.2)
Externally-orientated thinking	23.2 (5.2)	21.2 (4.3)
Total (α = .75)	68.7 (8.2)	63.8 (9.7)
*Waisman activities of daily living mean (SD) [Range]		
Total (α = .88)	28.0 (6.5) [14–34]	28.1 (5.4) [17–34]
^**$**^**PAT-A© session participation form (Assessment) mean (SD)**		
Verbal communication	2.9 (.7)	3.2 (.5)
Focus/ attentiveness	3.7 (.5)	3.8 (.4)
Understanding key concepts	3.4 (.6)	3.6 (.5)
Flexibility of thought	3.6 (.5)	3.4 (.8)
Speed of processing information	3.8 (.4)	3.8 (.5)
**Currently taking medication for a mental health condition N (%)**	13 (76.5)	12 (70.1)
**Previous experience of psychological therapy for a mental health condition N (%)**	14 (82.4)	15 (88.2)
**Previous inpatient admission for a mental health condition N (%)**	5 (29.4)	4 (23.5)

α = Cronbach’s alpha

*Higher scores indicate greater adaptive functioning, Max = 34

^$^Range = 1–4, lower scores suggest greater need for adjustments

#### Eligibility Criteria

##### Inclusion Criteria

Aged ≥ 18 years; diagnosed as autistic by an NHS clinical team; able to provide informed consent; with the verbal comprehension skills required to participate in interviews, talking therapy and questionnaire completion; and identified as experiencing clinically significant anxiety by an NHS clinician.

##### Exclusion Criteria

Not meeting the inclusion criteria; receiving current or recent (within three months) psychological therapies for anxiety; experiencing a mental or physical health condition that is likely to significantly affect capacity to engage in PAT-A. Participants were not excluded if they were receiving previous or ongoing pharmacological treatment for anxiety.

#### Recruitment

Clinicians approached eligible registered NHS patients and those interested returned an expression of interest form to the research team. Potential participants were sent written information by post or email depending on their preference. Those interested had a face-to-face meeting with the clinical research associate (CRA; SB) to discuss the trial, address questions or concerns, screen for eligibility and, if appropriate, proceed to taking written informed consent (participants had one week to consider taking part before deciding about participation).

### Measures

#### Baseline Characterisation

Measures completed at baseline and repeated as outcome measures are described below and in detail (including psychometric properties) in Parr et al. (2020). The first three measures were completed at baseline only and with participants’ informed consent, the ADIS-5 and PAIS-A© (see below) were audio-recorded to allow for inter-rater reliability to be assessed:

##### Anxiety and Related Disorders Interview Schedule for DSM-5: Adult Version (ADIS-5; Brown & Barlow, 2014)

A structured interview designed to identify current anxiety, mood, obsessive–compulsive, psychotic, trauma, and related disorders (e.g., somatic symptoms, substance misuse) according to DSM-5 criteria (American Psychiatric Association (APA), 2013). For each disorder indicated, the interviewer assigns a Clinician Severity Rating (CSR) based on the degree of associated distress and functional impairment. CSR ratings range from 0 to 8 whereby a CSR of ≥ 4 is considered to meet DSM-5 diagnostic threshold.

##### Personalised Anxiety Interview Schedule-Autism (PAIS-A; Brice et al., In Preparation)

Developed by the research team and administered alongside the ADIS-5. The PAIS-A© was designed to capture specific phenomena potentially related to anxiety experienced by autistic people (e.g., sensory processing differences, IU). As an example, there may be several reasons why an autistic person may be anxious about using public transport, including but not limited to concerns about being judged negatively by others, aversions to the sensory environment, or uncertainty (e.g., will the bus be on time?). The PAIS-A© aids clinical decision making about the relative contribution of such phenomena to anxiety in different situations. An addendum to the ADIS for autistic children has previously been developed and has demonstrated reliability and validity (Kerns et al., 2017).

##### A Few Things About Me for Therapy Interview Schedule

Developed by the Newcastle Neurodevelopment and Disability Team (https://research.ncl.ac.uk/neurodisability/leafletsandmeasures/therapyleaflets/afewthingsaboutmefortherapy/) was administered by the CRA after the participant had consented and used to assess which (if any) adjustments were necessary/desirable for the participant to increase the accessibility of trial processes and interventions.

##### The PAT-A© Session Participation Form (PATA-SPF)

A bespoke form developed for this study *and c*ompleted by the CRA after completion of baseline assessment and by the therapist after each treatment session. The PATA-SPF was used to identify ways to facilitate the participant’s participation in the assessment/ intervention sessions using five key markers, each rated on a four-point scale: (1) level of verbal communication, (2) focus/attentiveness, (3) understanding of key concepts, (4) ability to flexibly move between topics and (5) speed of processing information. This information was used by the clinical team to inform the provision of any adjustments required to support the participant’s engagement in future sessions.

Additionally, participants completed the following self-report questionnaires to enable characterisation of their autism profile, ability to understand and describe emotions, and adaptive functioning. Where available, outcome measures which have previously demonstrated acceptable psychometric properties for use with autistic adults were selected (see Parr et al., 2020 for full details). The internal consistency (Cronbach’s α) of the following baseline characteristic questionnaires is reported in Table 1.

- *Social Responsiveness Scale—2nd Edition *(*SRS-2; *Constantino & Gruber, 2012)*.* A standardised self-report questionnaire used to rate the social communication difficulties of autistic adults and children.
- *Toronto Alexithymia Scale-20 *(*TAS-20; *Bagby et al., 1994): a measure of alexithymia comprising of three subscales: difficulty describing feelings, difficulty identifying feelings, and externally-orientated thinking.
- *Waisman Activities of Daily Living Scale (W-ADL; *Maenner et al., 2013*)*: a measure of adaptive functioning and daily living skills, validated in people with a broad range of developmental disability diagnoses.

### Outcome Measures

#### Anxiety and Other Measures

The following questionnaires were completed by participants at baseline, and at three months post-intervention. The Anxiety Scale for Autism-Adult (ASA-A) and Hospital Anxiety and Depression Scale (HADS) were also completed immediately post-intervention. The internal consistency (Cronbach’s α) of the following outcome questionnaires is reported in Supplementary Table 6.

- *Anxiety Scale for Autism–Adult *(*ASA-A; *Rodgers et al., 2020)*.* A 20-item self-report questionnaire designed to measure anxiety in autistic adults (consists of a general anxiety factor and three group factors: social phobia, Anxious Arousal and Uncertainty). A total score of ≥ 28 on the ASA-A may indicate clinically significant anxiety.
- *Hospital Anxiety and Depression Scale *(*HADS; *Zigmond & Snaith, 1983)*.* A 14-item self-report questionnaire designed to measure symptoms of anxiety and depression.
- *WHO Quality of Life-BREF *(*WHOQOL-BREF; *World Health Organisation, 1998). A 26-item questionnaire covering four domains of quality of life (Physical health, Psychological health, Social relationships, and Environment). *The Disabilities module* (Power et al., 2010) and the *Autism Addendum *(*ASQoL;* McConachie et al., 2018) were also completed to assess disability-related and autism-related quality of life respectively.
- *EuroQoL 5 dimensions, 5 levels health survey (EQ-5D-5L;* Herdman et al., 2011). A generic measure of health-related quality of life.
- *Target Situation vignettes.* The participant and the CRA jointly agreed two important target situations associated with significant anxiety and impact on everyday functioning and QoL (e.g. going to the supermarket during a busy time). For both situations, a semi-structured interview was undertaken at baseline, 3 month and 12-month follow-up to determine the frequency and degree of anxiety, the participant’s response to the situation (symptoms and behaviour) and impact on daily functioning and QoL for the participant and people close to them. The CRA then summarised the information in a written vignette. All vignettes were anonymised and rated by researchers and clinicians, blind to intervention group. Using a well-recognised procedure with high levels of agreement between expert raters (Arnold et al., 2003; Maskey et al., 2014; Maskey et al., 2019a, 2019b, 2019c, 2019d), pairs of anonymised vignettes from baseline and 3 month follow-up were compared to identify whether there has been any change since baseline using a 9-point scale ranging from ‘very much improved’ to ‘disastrously worse’. Each vignette was rated by four panel members (unaffiliated to the study) independently and the mean score was calculated. The Target Situations interviews were repeated at 12 months post-intervention to investigate feasibility and acceptability.
- *Clinical Global Impression of Improvement scale *(*CGI-I;* Busner & Targum, 2007)*.* A standardised framework for rating to what extent the participant’s symptoms have improved or worsened on a 7-point scale ranging from ‘very much improved’ to ‘very much worse’. At 3 months post-intervention, CGI-I ratings were completed by an independent trained researcher blind to intervention group using anonymised baseline demographic information and ADIS-5 diagnoses with CSR ratings; together with baseline and 3 month follow-up data for the following measures: Target Situation vignettes and mean change scores; item-level data from the ASA-A and HADS; full scale and subscale scores from the ASA-A, HADS, WHOQoL-BREF plus disabilities module and ASQoL and EQ-5D-5L. Qualitative information about any change to anxiety-related situation(s) not already covered in the target situation vignettes.

#### Acceptability

Once all the 3-month post-intervention follow up measures had been completed, participants were offered an opportunity for a semi-structured interview. The interview focused on the acceptability of trial methods and materials, and the PAT-A© or CCSP interventions. Participants were also asked about any changes (positive/negative) in any anxiety-related situation not covered by the target situations.

### Procedures

Prior to the onset of the COVID-19 pandemic, all measures/interviews were completed face-to-face with the trained CRA at a location previously agreed with the participant (usually their home or a local NHS clinic). From March 2020, interviews were completed by telephone, video call, or text-based chat (according to participant preference). Questionnaires were completed online via Qualtrics (Qualtrics, 2005) because sending/returning paper questionnaires by post did not meet UK Government COVID-19 restrictions definition of ‘essential travel’.

### Formulation and Treatment Planning

Once all baseline measures had been completed and prior to randomisation, the CRA compiled a standardised report for each consented participant (including demographic information; information on anxiety disorders and other diagnoses (with CSR rating and key mechanisms identified using the PAIS-A©), adjustments potentially required and key information from baseline measures such as alexithymia and autism ‘profile’). Each report was then discussed at a meeting of clinicians within the trial team. The CRA was available to address any questions but left the meeting before treatment planning decisions were made to ensure CRA remained blind to clinical decision and formulation. An anxiety-specific formulation (a theory-based explanation of the origins, development and maintenance of presenting problems that guides intervention) was then agreed and a *bespoke treatment algorithm procedure (*designed by the research team) used to inform the individualised treatment plan. The treatment algorithm procedure provided a decision tree in which the clinical formulation of each participant’s overall anxiety presentation informed decision-making regarding choice of treatment modules. Firstly, the clinical team identified the most pertinent factor(s) underlying the participant’s anxiety presentation (e.g., IU, social evaluation, specific situation, etc.). The algorithm then guided the use of other relevant information to inform clinical decision-making (e.g., whether the anxiety occurred across situations or in a specific situation; whether the threat was internal or external; whether factors such as sensory processing differences were relevant; the participant’s readiness for change) (see Supplementary Fig. 1 for an illustrative example whereby IU is the most pertinent factor underpinning the participant’s anxiety experiences). To investigate reliability, the CRA used the algorithm (independent of the other clinicians involved in formulation) to create a proposed treatment plan for each consented participant and sealed this in an envelope. At the end of the team meeting the two treatment plans were compared and agreement recorded. In the event of disagreement, the decision of the clinical team was final. For participants randomised to PAT-A© intervention, any new clinical information that arose during therapy was reviewed during supervision and if needed a further clinicians meeting could be organised to review the treatment plan. The CRA remained blind to this procedure.

### Randomisation

All consented eligible participants were randomised following the formulation and treatment planning meeting to either the intervention group (PAT-A©) or the control group (CCSP) via computer-generated sequence blocks using Sealed Envelope; http://www.sealedenvelope.com/ (simple randomisation schedule without blocking or stratification). An independent research administrator oversaw this process. A trial therapist informed participants of the randomisation outcome and intervention status and recorded this in their NHS electronic patient record.

### Intervention

#### Personalised Anxiety Treatment-Autism (PAT-A) (Intervention Group)

PAT-A© is a new intervention comprising five manualised modules designed specifically for use with autistic people (see Supplementary Fig. 2). Each module is designed to stand-alone but can be used in combination. All modules include key techniques of CBT (e.g. agenda setting, guided discovery, and ‘homework’ setting). Based on the individualised formulation and treatment plan, participants randomised to the intervention group were offered a bespoke intervention package of two or more of the following PAT-A© modules:

#### Understanding and Describing Emotions (UaDE)

UaDE aims to develop a person’s ability to understand their own emotional states and how different situations may be associated with different emotions. In addition, there was some limited psychoeducation on behavioural coping strategies to help manage distress (e.g. muscle relaxation, positive visual imagery). All participants randomised to PAT-A© intervention were offered UaDE as the preliminary module (the number of sessions depended on the participant’s pre-existing knowledge and ability in relation to recognising and communicating emotions) prior to receiving additional PAT-A© treatment modules as part of the treatment plan.

#### Mindfulness

This module aims to provide skills to enable tolerance of emotional distress in everyday situations and/or facilitate engagement with anxiety-provoking situations and behavioural experiments outlined in other PAT-A© treatment modules. The Mindfulness module was designed to offer either a briefer ‘Mini-mindfulness’ approach (2–3 sessions) to support emotion regulation or a full approach (6–8 sessions) depending on the individual’s needs.

#### Coping with Uncertainty in Everyday Situations (CUES-A©)

The focus of the CUES-A© intervention is to support participants in developing awareness and confidence in their ability to recognise and manage their IU through collaborative CBT strategies. See Rodgers et al. (2018) for a full description.

#### Modified CBT for Social Anxiety

Autistic adults may experience fear and anxiety in social situations for a variety of reasons. This module is designed for those where social evaluative concerns are the predominant difficulty (i.e., reflecting DSM-5 social anxiety disorder). It was developed specifically for use in PAT-A©, and contains the main elements commonly used in recommended treatments for social anxiety (NICE, 2013) that have been adapted for effective use with autistic adults (e.g., assessment and consideration of social communication difficulties).

#### Immersive Virtual Reality Environment-Delivered Graded Exposure Treatment (VRE) for Phobias, and Situational Anxiety

VRE uses visual images projected onto a screen (with sound) over four 20-min sessions. Participants interact with and navigate through graded exposure in relation to a specific phobia scenario using a tablet with trained therapist support (see Maskey et al., 2019a, 2019b, 2019c, 2019d) for a full description). Due to COVID-19 and treatment being delivered in 2020, it was not possible to use the VRE as planned, and thus CBT delivered by a therapist took place in real life settings, with use of appropriate Personal Protective Equipment and appropriate consideration of adjustments to graded exposure that were specific to the participants needs (e.g. sensory differences).

#### Current Clinical Services Plus (CCSP; Control Group)

Participants randomised to the control group were offered two standardised psycho-educational sessions based on the UaDE intervention module, focussing on understanding and describing emotions alongside information on basic coping strategies to help manage distress. They were then signposted (and referred if necessary) to NHS services available as part of usual care.

### PAT-A© Therapist Training and Treatment Fidelity

PAT-A© was designed to be delivered by clinicians with appropriate training in CBT (e.g. clinical psychologists or psychological therapists with accredited CBT training) and experience working with autistic people. Therapists were trained in delivering all PAT-A© modules, received individual fortnightly supervision and monthly group supervision (from one of the co-authors, BI). They also had access as required to the trial team (comprising experts with complementary expertise in CBT and adapting psychological therapies for autistic people).

A bespoke *Session Recording Form* (PATA-SRF) was designed to assess aspects of fidelity of delivery of the PAT-A© intervention (e.g. recording whether core CBT techniques were used in each session, and tracking treatment module selection and whether specific components of each module were delivered within a session as per the treatment manual). The therapist completed the PATA-SRF after every session. Where acceptable to the participants randomised to the PAT-A© arm, therapy sessions were audio recorded.

### Analyses

Analyses were conducted in accordance with the pre-specified statistical analysis plan. Recruitment and retention rates together with rates of engagement with PAT-A© intervention(s) were analysed descriptively. Participants’ experiences regarding the acceptability of PAT-A© intervention, CCSP (control group), and trial methods and materials were analysed using the qualitative data collected during the acceptability interview. Primarily yes/no answers (e.g. was the amount of time that it took to complete questionnaires acceptable?) were analysed descriptively. Thematic analysis based on Braun and Clark’s (2006) framework was used to analyse data from open-ended questions (e.g. discussing the strengths and limitations of the PAT-A© approach). Qualitative data were analysed by SB and CW before themes were jointly identified through discussion.

Relating to trial feasibility, the inter-rater reliability of several key aspects of the trial was assessed by comparing scores between two independent trained researchers blind to treatment allocation. This included examining the inter-rater reliability of the ADIS-5/PAIS-A©, the treatment planning algorithm and CGI-I ratings. Analysis was either descriptive (CGI-I and algorithm) or by calculating the intraclass correlation coefficient (ICC; ADIS/PAIS-A© and PATA-SRF). Data from completed PATA-SRF forms (as a proxy for treatment fidelity) were analysed descriptively to assess fidelity of therapists’ delivery of the PAT-A© intervention.

Change between baseline and follow up on target situation vignettes (3 months and 12 months post-intervention) and CGI-I (3 months post-intervention) was analysed descriptively. For both Target Situations and the CGI-I, frequencies of participants rated in each category of change (improved, no change, worsened) are reported by group. For questionnaires completed at baseline and follow up (immediate or 3 months post-intervention) change was analysed descriptively (means and standard deviations) at full-scale and, if appropriate, subscale level.

### The Involvement of Autistic People and Relatives in this Research

This research is directly aligned to published research priorities of the autism community (James Lind Alliance, 2016). Autistic people were members of the research team and involved from the outset in the trial design. A relative of an autistic adult advised on trial methods and procedures and supported discussion groups with autistic people who reviewed and advised on trial materials. Autistic people were involved in developing the PAT-A© intervention, data analysis and interpretation, dissemination, and manuscript preparation. Our dissemination plan includes effective and accessible ways to share the findings with autistic people.

## Results

Demographic information presented in Table 1. Thirty-four participants were consented and randomised to the trial, of these 65% in each trial arm were male. The PAT-A© group were on average 6.4 years older than the CCSP group. The mean SRS-2 score was similar for each group (PAT-A© = 125.1; CCSP = 110.1) and well above the autism cut-off score of 67 (Constantino & Gruber, 2012). The PAT-A© group received their autism diagnosis mean 6.7 years later than the CCSP group. Current and prior experience of anxiety medication and psychological therapy for anxiety were similar for both groups, as were the presence of other co-occurring physical and mental health conditions.

### Recruitment and Retention

**Fig. 1:**
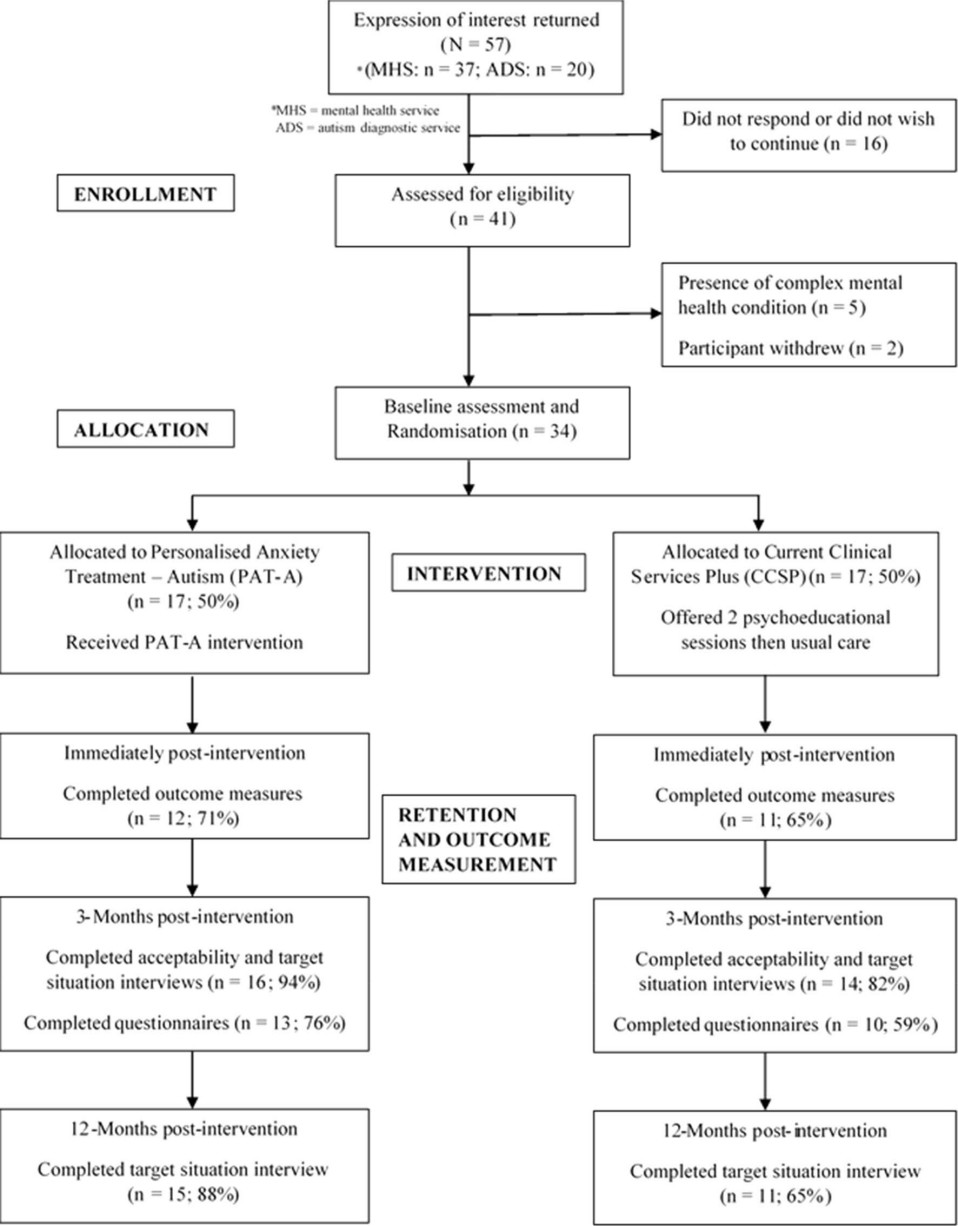
CONSORT flow diagram

All participants were recruited between November 2018 and October 2019 via NHS clinical services [community mental health services (including psychological therapies services) and adult autism assessment & diagnosis services]. Information regarding the rates of recruitment, retention and follow up can be found in Fig. 1.

Seventeen participants were randomised to PAT-A©; 14 completed their treatment, 3 withdrew. Of those who terminated treatment early, one attended two sessions and did not give reason for withdrawal or complete follow up outcome measurement. Two participants ended treatment early but provided follow up data: one after 3 sessions (no longer wished to continue), the other after 8 sessions in mutual agreement with the therapist due to difficulties relating to other (non-anxiety) mental health conditions. Four participants randomised to PAT-A© were in active treatment in March 2020 when Covid-19 pandemic restrictions were enforced.

Of the participants (N = 17) randomised to CCSP, one disengaged following dissatisfaction at the outcome of randomisation, withdrew consent for retention of baseline questionnaire data and did not provide follow-up data; 16 were offered two psychoeducational sessions. Twelve participants attended both sessions; 2 attended one session (home schooling a child and no reason given) and 2 attended neither session (problems getting to NHS premises and a family member in ill health). Support to attend sessions was offered but not accepted.

One participant in the PAT-A© group was lost to follow up (after disengaging from treatment) alongside three from the CCSP group. All other participants completed at least one follow up. At three months post-intervention follow up, half the sample (PAT-A© n = 5; CCSP n = 10) completed face-to-face assessments prior to introduction of COVID-19 restrictions in March 2020; the remaining participants (PAT-A© n = 11; CCSP n = 4) completed measures remotely. Eighty seven percent of those whose 3-month post intervention follow up took place prior to the pandemic completed all questionnaires, compared to 67% of those assessed remotely. All assessments completed 12 months post-intervention were completed remotely.

All participants met criteria for at least one anxiety disorder. The median number of current anxiety disorder diagnoses was 3 in each study arm. The mental health diagnoses of participants, as well as information about co-occurring physical health conditions is shown in Supplementary Table 1. A clinical team member (PW) independently listened to recorded assessments of eight participants (23.5%) to assess inter-rater reliability of the ADIS-5 and PAIS-A©. A two-way random model using absolute agreement showed ‘good’ inter-rater reliability (Intraclass Correlation Coefficient; ICC = 0.817). Taking each participant’s most severe anxiety disorder (anxiety disorder with the highest Clinician Severity Rating (CSR)), the median severity score in both groups was 6 (8 is the highest score; 4 the clinical cut-off).

### Treatment Allocation

Treatment plan agreement between the CRA and wider team was: Full agreement (n = 20, 59%); partial agreement n = 11, 32%); no agreement (n = 3, 9%). In the 3 cases where there was no agreement, the participants’ anxiety presentations were affected by several factors (e.g., IU and sensory processing differences) meaning that there were several potentially valid treatment options. The frequency that each treatment module was indicated as a primary or secondary module is shown in Supplementary Table 2. Of the 17 participants in the PAT-A© group, 4 (24%) were subsequently reformulated (two in light of clinical changes as treatment progressed and two in response to changes relating to COVID-19). Of the participants who completed their treatment and had available data relating to the content of sessions they received (N = 12), four (33%) received at least one session from one module additional to UaDE, four (33%) across two additional modules, and four (33%) across three additional modules. In this group, eight participants received 12 sessions, three received 11 sessions and one received 8 sessions.

### Fidelity to the PAT-A© Intervention

Recorded PAT-A© clinical sessions were available for nine participants (52.9%). The number of recorded sessions ranged between 3 and 6. Some participants did not consent to their sessions being audio recorded and it was not feasible in other cases (e.g., if sessions took place outside the clinical environment). To investigate whether the PATA-SRF was a reliable measure of feasibility and adherence to the manualised PAT-A© intervention, the CRA independently coded all recorded sessions from two randomly selected participants (N = 7 sessions). Inter-rater reliability was ‘good’ (ICC = 0.858). Descriptive data relating to the extent to which therapists self-reported adherence to the essential components in each of the treatment manuals used, as well as their use of core CBT skills can be seen in Supplementary Table 3. These data show that the majority of PAT-A© sessions in each module addressed at least one essential element of the intervention. There is more variability regarding the extent to which every essential element of each module was addressed, partly because some participants only received one or two sessions from a specific module (based on clinical need).

### Acceptability of Research Processes

Participants who completed the acceptability interview at 3 months post-intervention follow up (N = 30; 94% of PAT-A© group; 82% of CCSP) were asked about the acceptability of research processes. Table 2 shows participants’ responses to primarily yes/no questions (e.g., “was the length of time that it took to complete questionnaires acceptable to you”?). Analysis of more qualitative responses (e.g., “what (if anything) would you change about the intervention(s)”?) is presented separately. Many commented that the baseline anxiety interview (i.e., ADIS-5 and PAIS-A©) allowed them to ‘feel listened to’ and furthered their understanding of anxiety and autism and for some was ‘the start of their therapeutic journey’. Several participants commented that the baseline interviews were long and sometimes ‘draining’ but appreciated that it could be completed over more than one session. Most aspects of trial methodology were acceptable to more than 90% of the sample. Eighty one percent of the PAT-A© sample, and 86% of the CCSP sample agreed they had enough information prior to taking part and PAT-A© one participant commented that initial study information they received was difficult to understand due to the language used. Identifying two anxiety-related situations was acceptable to 75% of the PAT-A© sample and 93% of the CCSP sample. Two participants found it difficult to identify and/or describe anxiety related situations, one stated that they wished the researcher had ‘taken the lead’ in proposing a situation and one suggested that these situations (established at baseline) felt too difficult to achieve once therapy had commenced.

**Table 2:** The acceptability of key aspects of the research process to participants

	N (%) reporting unequivocal acceptability	
	PAT-A© (n = 16)	CCSP (n = 14)
Did you have enough information about the study prior to the first meeting with the research team?	13 (81.3)	12 (85.7)
Did you have opportunity to ask questions and/or discuss concerns with the research team prior to consenting?	15 (93.8)	14 (100.0)
Was the length of the diagnostic interview at baseline acceptable?	15 (93.8)	14 (100.0)
Was coming up with two personal anxiety related situations acceptable?	12 (75.0)	13 (92.9)
Was the amount of time that it took to complete the questionnaires acceptable?	15 (93.8)	14 (100.0)
Did you understand what it meant to be randomised and was the justification for randomisation clear?	15 (93.8)	14 (100.0)

### Acceptability and Impact of the Interventions

#### PAT-A© (Intervention Group)

Sixteen participants responded to open ended questions about what they thought went well about the PAT-A© treatment that they received, what did not go so well, and what they would have changed. Four themes emerged following thematic analysis:

1. *Transferrable skills*. Many participants commented that the skills they learned in sessions were allowing them to manage anxiety in daily life.

I learnt some new techniques for managing anxiety and dealing with negative thoughts…They are useful techniques that work. I feel like I could draw on these skills on a daily basis” and “There was quite a lot of looking at specific things that bothered me, and using the same techniques to deal with them, and that really helped me to spot the patterns after the sessions stopped.

2. * Adjusted to meet autistic people’s needs*. Several participants thought the intervention and therapists’ approach was set up to suit them (e.g., communication preferences).

[Therapist’s name redacted] would reword stuff in a different way for an autistic person so she would keep on asking questions, and asking them in a different way, and making sure I understood everything fully. Whereas before when I have had therapy people just expect you to understand stuff.

3. * Positive impact of PAT-A©*. Many participants experienced improvements in their anxiety symptoms and quality of life following completion of PAT-A©.

I have had therapy since I was 17, and this was by far the best therapy I have had…it has helped me so much in such a short space of time. I have grown so much and learnt so much about myself.” And “Everything is good now, my outlook on everything is different. I have made massive changes to my life…Everything is an option now.

4. *More sessions needed*. Of those who would have changed something about PAT-A©, most commented that they would have preferred to have more sessions.

I think it would have been helpful to having some continuity with [the therapist] or another service. For me it was a big change going from sessions to no sessions.

#### CCSP (Control Group)

One participant allocated to CCSP withdrew from the study due to randomisation outcome and another participant expressed they did not think there should be a control group. Several CCSP group participants expressed that the two psychoeducational sessions were of limited help and similar to support received in the past. However, many commented they felt glad to be a part of research and some reported that the two sessions were helpful. Several participants (across both arms) commented that they “felt part of something” and hoped that by taking part, they were supporting widening access to effective anxiety treatments for autistic people.

### Outcome Measurement

#### Target Situation and Global Clinical Impression of Improvement (CGI-I)

Thirty-three participants chose two anxiety related situations, and one participant chose one situation. Participants identified a wide range of personally salient anxiety related contexts at baseline. The situations chosen most commonly related to: engagement with activities (e.g., hobbies, interests, employment/education, etc., N = 18 vignettes); attending to public places/events (e.g., supermarkets, restaurants, parks, shops, public transport, etc., N = 18); socialising with family or friends (N = 17); communicating with others (N = 8).

At the three months post-intervention follow-up, in addition to the four participants who did not complete the follow up interview for Target Situations, some information to inform vignettes could not be collected due to the impact of COVID-19 restrictions (e.g., Target Situation was to eat a meal in a restaurant, but restaurants were closed at the time of follow up). This meant there were several vignettes that could not be completed (five vignettes in the PAT-A© group and three in the CCSP group). Descriptive data showing the mean (of four independent raters) Target Situation vignette rating change from baseline to three months post-intervention can be seen in Supplementary Table 4.

Twenty two percent (N = 5) of CGI-I ratings were independently coded by a senior member of the research team (JR; blind to group). Independent raters had full agreement on 80% of CGI-I ratings and were within one point on the remaining 20%. Descriptive data relating to the CGI-I change ratings from baseline to three months post-intervention is shown in Supplementary Table 5. Of those participants eligible to be rated in the PAT-A© group, 23% were clinically ‘much improved’ and 39% ‘minimally improved’, compared to 30% and 0% respectively in CCSP. No participant in either group was rated to have got ‘much worse’ or ‘very much worse’ at follow up, but some were rated as being ‘minimally worse’ (PAT-A: 23%; CCSP: 40%).

#### Questionnaire Data

The return rates of questionnaire data were affected by the COVID-19 pandemic. Some participants suggested they would not be willing or able to complete the questionnaires over the telephone or on a screen. Descriptive data showing mean subscale and total scores on all outcome measures completed at baseline and immediately and three months post-intervention is shown in Supplementary Table 6. Data show that mean anxiety (measured by ASA-A and HADS-A) reduced from baseline to immediately post-intervention in both groups. There is score variability both within and between groups with wide standard deviation. There was no substantial change in QoL between baseline and 3-month follow-up in either group.

## Discussion

This is the first report of an RCT of a new bespoke modular anxiety treatment for autistic adults (PAT-A©) and to our knowledge, the first time such an approach to treating anxiety experienced by autistic people has been developed and manualised. The findings show that it was feasible to recruit eligible autistic adults via NHS clinical services and NHS clinicians were keen to refer. Participant retention and attrition were comparable to other similar feasibility RCTs in this population (Russell et al., 2020), including in the control group where uptake of the psychoeducational sessions was good. Autistic adults were willing to be recruited and randomised and many reported that they viewed their participation as an opportunity to help make effective anxiety treatments available to future generations of autistic people.

A significant majority of participants reported that key aspects of trial design (including randomisation) and materials (including selected outcome measures) were acceptable to them. A smaller proportion of participants completed questionnaires at follow up than those who completed interviews at the same time point, although questionnaire return rate was in line with other recent research (e.g., Russell et al., 2020). It appears likely that restrictions associated with the UK government’s response to COVID-19 (i.e. all assessments needed to be completed remotely) impacted on response rates, with a 20% drop in questionnaire response rates for those interviewed during the pandemic compared to those whose follow up pre-dated the pandemic. Whilst some participants were not able to complete questionnaires online or remotely, others appreciated the opportunity to do so, suggesting that a future trial should adopt a flexible approach to data collection procedures. A quarter of participants in the PAT-A© group had some difficulty identifying two target anxiety-related situations compared to 7% in the CCSP group. Some participants offered insights as to why this might be, suggesting that target situations may have to be more flexible to account for treatment goals that change or evolve in the course of treatment. The CGI-I appears to be a reliable measure and allowed for multiple sources of information to inform outcome assessment. Additionally, the CGI-I (and therefore outcome measures considered in its calculation) appear to demonstrate sensitivity to change in this trial, with ratings ranging from slightly worse/no change to much improved. We propose that this should remain as the primary outcome measure in any future trial of PAT-A©.

Whilst this study was not designed as a fully powered trial, the occurrence of the COVID-19 pandemic during the course of this study conferred additional challenges to the interpretation of available outcome data. Half of all participants completed their 3 month follow up during the pandemic but due to the difference in the length of interventions, this included a disproportionate number of participants randomised to the PAT-A© group (69% of PAT-A© during COVID-19 compared to 29% of CCSP). Several studies have indicated the COVID-19 pandemic has impacted significantly on the mental health of autistic people (Bundy et al., 2022), meaning that any comparisons with anxiety measures that occurred before the pandemic are likely confounded by the considerable changes in contexts as a consequence of the onset of the pandemic. The findings from the qualitative interviews provided additional information about context and attitudes to both interventions. Participants supported the concept of a personalised, modular psychological intervention and felt that the PAT-A© approach was well adapted to their needs and accounted for autism. The qualitative thematic analysis also provided some preliminary evidence that PAT-A© led to positive, real-world changes to anxiety and daily life. In our opinion, a future trial would benefit from further qualitative work with participants in both intervention groups as part of a mixed-methods analysis plan.

This sample presented with complex and pervasive anxiety difficulties, consistent with other studies (Joshi et al., 2013) as well as other co-occurring physical and mental health conditions. When considering each participant’s most severe CSR rating, the median in each group was six, indicating severe anxiety likely to impact significantly on daily life. The experience of multiple anxiety disorders was common, with 71% of the PAT-A© group and 65% of the CCSP group meeting threshold for three or more concurrent DSM-5 anxiety disorders. This supports the need for and importance of a modular approach to intervention that extends beyond treating one specific anxiety disorder. Interestingly, approximately a third of participants in both groups experienced anxiety categorised as ‘Other Specific Anxiety Disorder’ because their presentation did not meet current diagnostic criteria for a specific DSM-5 anxiety disorder. These findings add support to the recommendation that for autistic individuals, an expectation of the possibility of one or more anxiety disorders and a focus on aspects of the autism profile (such as IU, sensory processing differences) need to be carefully considered in the assessment and treatment of anxiety symptoms and presentations (e.g.,; Moore et al., 2021; South & Rodgers, 2017).

### Strengths and Limitations

The research is in direct response to the research priorities of the UK autism community (James Lind Alliance, 2016). It is a methodologically strong single blind feasibility and acceptability RCT that included a control group of current best practice and a novel individualised treatment using a manualised set of five intervention modules, an algorithm for planning treatment and standardised assessment measures. The PAT-A© intervention appeared to be delivered with fidelity and the newly developed PATA-SRF demonstrated good inter-rater reliability. One challenge with the PATA-SRF that may require amendment in the future was that dose (i.e. the number of sessions a participant received of a particular module) could confound fidelity. For example, therapy with a participant who only received a short social anxiety intervention as part of a multi-module treatment plan would not reasonably be expected to cover all essential elements of the module compared with another participant who received a longer social anxiety intervention. Whilst we have developed a methodology for recording the dose and content of a personalised PAT-A© intervention, it would be interesting to explore in a future trial how these considerations may impact on the acceptability and efficacy of PAT-A©. Our findings provide early evidence for the proof of concept and reliability of this new manualised modular intervention, the use of standardised measures and bespoke measures.

The complexities of the COVID-19 pandemic provided additional unexpected challenges to the delivery of both interventions, the research procedures and interpretation of the findings. The UK Government regulations, for example, restricted our ability to offer a VRE intervention as planned, and led to the use of non-face to face follow-up assessments including the use of telephone calls and on-line and other virtual methods for completion of outcome measures. In the wake of the pandemic it is likely that more flexible approaches to delivering interventions and assessment measures will need to continue and the advantages and disadvantages of different modes of delivery will require further evaluation.

The PAT-A© intervention is designed to offer treatments for the most common anxiety subtypes experienced by autistic people and target key mechanisms. However, there may be a need to incorporate additional modules in the future. For example, a significant percentage of our sample met criteria for a panic disorder. Although our existing modules (e.g. mindfulness) provide reasonable treatment options, there is potential to incorporate a specific module for panic. Further work would be needed to adapt or develop such a module for autistic people. Finally, the use of Target Situations in its current form appears to have lacked the flexibility to account for treatment goals that evolve in the course of treatment (and/or in response to unexpected changes in context). This measure will need to be reviewed in the design for a definitive clinical and healthcare efficacy and cost effectiveness RCT.

### Clinical and Research Implications and Future Directions

A flexible and individualised manualised approach to treating anxiety experienced by autistic people shows promise, especially given the experience of multiple concurrent anxiety disorders in this sample and other research (e.g., Joshi et al., 2013). In this trial, PAT-A© was able to be delivered by clinicians with CBT skills and experience of working with autistic people. The clinicians only required brief additional training and access to regular supervision equivalent to current clinical practice. As such, it is likely that PAT-A© could feasibly be used in clinical services with limited additional training (e.g., IAPT; secondary mental health care). Implementation would require the availability of assessment measures that are valid and reliable in reflecting autistic people’s anxiety experience. We will seek to gather further evidence regarding the psychometric properties of the bespoke assessment measures used in this trial alongside developments from other research teams and clinical practice. The evidence from this feasibility and acceptability trial will also inform planning for a definitive trial.

Further development and refinement of treatments for anxiety experienced by autistic people are to be welcomed. In keeping with the findings from this research and other published studies, recognition of the range of anxiety disorders, the relevance and importance of co-occurring conditions such as other neurodevelopmental skills and needs, and the likelihood of additional mental health and indeed physical health difficulties also need to be considered in a holistic approach to mental health treatment for autistic people. Further information regarding PAT-A© and new measures (e.g., the PAIS-A©) will be published separately. Future developments of PAT-A© to assessment and intervention planning could include consideration of the needs of autistic children, adolescents, and their families, through to modular approaches for older autistic people. In light of recommendations for stepped care approaches across time (see Green et al., 2022), work should be undertaken with NHS clinical services to explore and respond to barriers implementing personalised and flexible anxiety interventions for autistic people.

## Conclusions

The PAT-A© intervention approach, as well as trial methods and materials, were acceptable to autistic adults and feasible to implement. Whilst it is not possible to reliably report on the efficacy of PAT-A© due to the planned lack of statistical power of this RCT-pilot study and the unexpected changes relating to the COVID-19 pandemic, there is early evidence to suggest that PAT-A© can lead to real world reductions in anxiety and improvements in daily life. A definitive trial is required to assess the clinical and cost-effectiveness of PAT-A©. In addition, further development of bespoke assessment measures and treatment modules is advised and there is a need to explore and address potential barriers to implementation in the UK NHS.

## Supplementary Information

Below is the link to the electronic supplementary material.

Supplementary file1 (DOCX 149 KB)

## Data Availability

Reasonable requests for additional information and/or anonymised data can be submitted to the corresponding author. Materials and training will be available in the future. To register interest, please contact the corresponding author jacqui.rodgers@ncl.ac.uk.
