# Supplementary material for: A Pilot Randomised Control Trial Exploring the Feasibility and Acceptability of Delivering a Personalised Modular Psychological Intervention for Anxiety Experienced by Autistic Adults: Personalised Anxiety Treatment-Autism (PAT-A)
Source: J Autism Dev Disord. 2023 Sep 20;54(11):4045–60. doi: 10.1007/s10803-023-06112-5 (PMC11461680; doi:10.1007/s10803-023-06112-5)
Supplement: Supplementary file 1 — Supplementary file1 (DOCX 149 KB) [file 10803_2023_6112_MOESM1_ESM.docx]

**Supplementary Figure 1. Example of how the treatment algorithm informs clinical decision-making when the primary anxiety mechanism is difficulties coping with uncertainty**

**
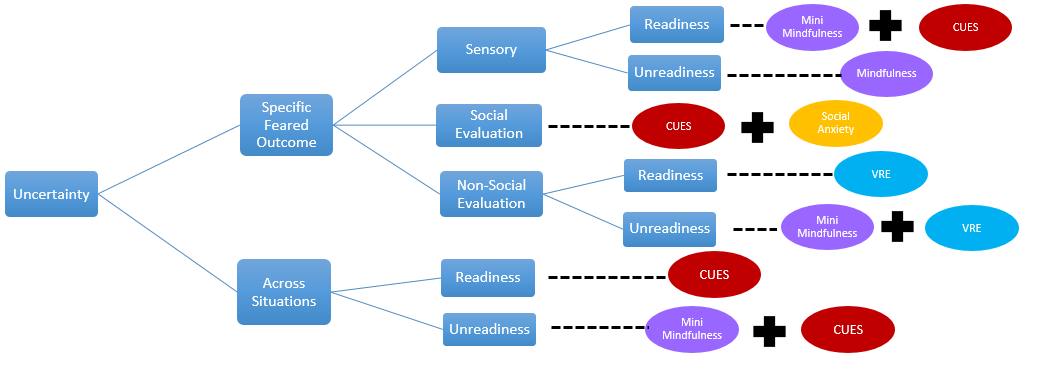
**

*Readiness/ Unreadiness refers to the extent to which the participant is judged by the clinician to possess the requisite skills (e.g., recognising, communicating, and regulating emotions) to engage in a CBT-based treatment (e.g., exposure and response prevention).*

*CUES: Coping with Uncertainty in Everyday Situations; VRE: Virtual Reality Environment augmented treatment for phobia and situational anxiety.*

**Supplementary Figure 2. Personalised Anxiety Treatment – Autism (PAT-A©) treatment modules**


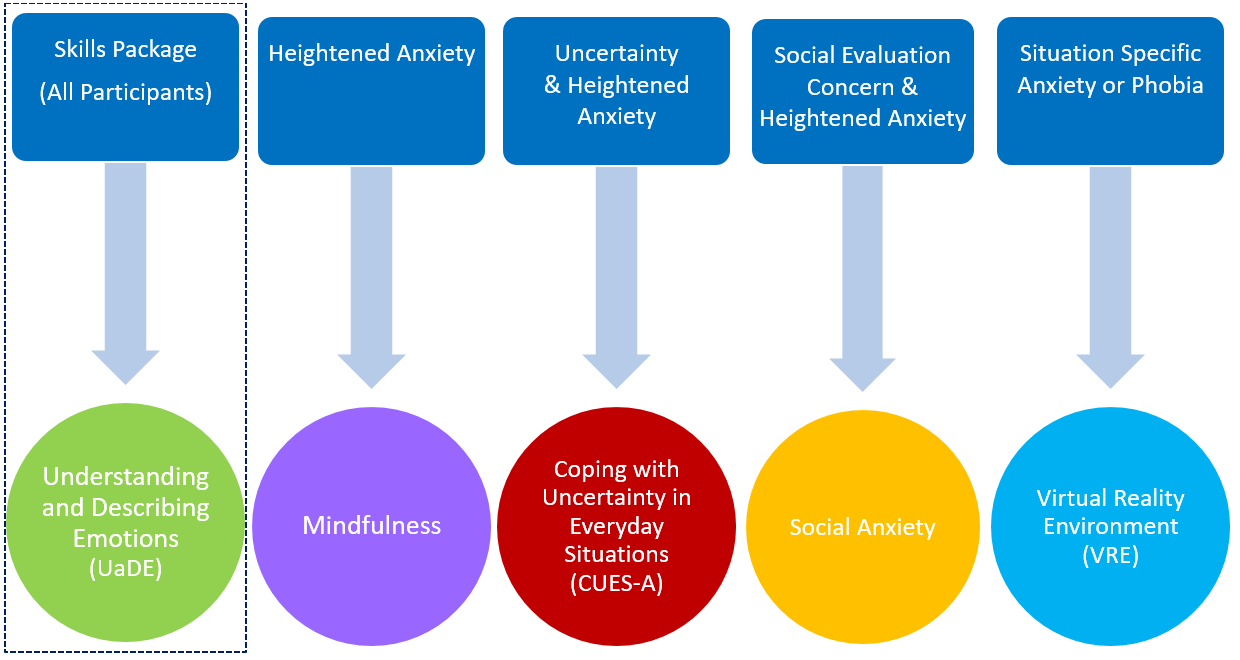


**Supplementary Table 1. ADIS-5 anxiety and co-occurring mental health diagnoses and physical health conditions**

| **Diagnosis** | **PAT-A** | **CCSP** |
| --- | --- | --- |
| **Anxiety Disorders** N (%) |  |  |
| Generalised Anxiety Disorder | 15 (88.2) | 12 (70.6) |
| Social Anxiety Disorder |  |  |
| Social Anxiety Disorder | 13 (76.5) | 15 (88.2) |
| Other Specified Anxiety Disorder (Social) | 3 (17.6) | 2 (11.8) |
| Any Social Anxiety | 16 (94.1) | 17 (100.0) |
| Specific Phobia | 10 (58.8) | 10 (58.8) |
| Separation Anxiety |  |  |
| Separation Anxiety Disorder | 4 (23.5) | 3 (17.6) |
| Other Specified Anxiety Disorder (Separation) | 2 (11.8) | 1 (5.9) |
| Any Separation Anxiety | 6 (35.3) | 4 (23.5) |
| Panic Disorder |  |  |
| Panic Disorder | 1 (5.9) | 2 (11.8) |
| Panic Disorder with Agoraphobia | 1 (5.9) | 2 (11.8) |
| Other Specified Anxiety Disorder (Panic) | 4 (23.5) | 3 (17.6) |
| Any Panic | 5 (29.4) | 7 (41.2) |
| Agoraphobia | 3 (17.6) | 2 (11.8) |
| **Anxiety Comorbidity**  N (%) |  |  |
| At least two anxiety diagnoses | 17 (100.0) | 16 (94.1) |
| At least three anxiety diagnoses | 12 (70.6) | 11 (64.7) |
| At least four anxiety diagnoses | 7 (41.2) | 6 (35.3) |
| Median number of anxiety disorder diagnoses | 3 | 3 |
| At least one atypical anxiety disorder (i.e. ‘other specified’) | 6 (35.3) | 6 (35.3) |
| **Other Mental Health Disorders**  N (%) |  |  |
| Depression |  |  |
| Major Depressive Disorder | 6 (35.3) | 1 (5.9) |
| Persistent Depressive Disorder with Major Depressive  episodes | 0 (0.0) | 5 (29.4) |
| Persistent Depressive Disorder | 7 (41.2) | 5 (29.4) |
| Any Depressive Disorder | 13 (76.5) | 11 (64.7) |
| Obsessive Compulsive Disorder | 5 (29.4) | 3 (17.6) |
| Body Dysmorphic Disorder | 2 (11.8) | 5 (29.4) |
| Post-Traumatic Stress Disorder | 0 (0) | 2 (11.8) |
| Somatic Symptom Disorder | 0 (0.0) | 1 (5.9) |
| **Other Mental Health Disorder Comorbidity**  N (%) |  |  |
| At least one other mental health diagnosis | 14 (82.4) | 11 (64.7) |
| At least two other mental health diagnoses | 5 (29.4) | 8 (47.1) |
| Median number of other mental health diagnoses | 1 | 1 |
| **Co-occurring Physical Health Conditions**  N (%) |  |  |
| At least one physical health condition | 8 (47.1) | 6 (35.3) |
| At least two physical health conditions | 3 (17.6) | 3 (17.6) |

**Supplementary Table 2. PAT-A© treatment modules and the frequency to which they were indicated as a primary or secondary treatment module during the formulation and treatment planning procedures.**

| **Treatment Module** | **Primary**  N (%) | **Secondary** N (%) |
| --- | --- | --- |
| UaDE*** | 2 (5.9) | 0 (0.0) |
| Mindfulness | 5 (14.7) | 1 (2.9) |
| Mini-mindfulness | 8 (23.5) | 0 (0.0) |
| CUES | 9 (26.5) | 12 (35.3) |
| Social Anxiety | 10 (29.4) | 9 (26.5) |
| VRE Situation Specific Anxiety | 0 (0.0) | 4 (11.8) |

** All participants received UaDE, for those included in this table, UaDE was indicated as a particularly important aspect of the participant’s treatment in their formulation*

**Supplementary Table 3. The extent to which therapists self-reported adherence to the general concepts of CBT and essential components of each PAT-A© module**

|  |  | **Number of sessions each participant had per module** | **Percentage of sessions that addressed at least essential element of the module to a considerable degree** | | **Percentages of interventions that were addressed to a considerable degree** | |
| --- | --- | --- | --- | --- | --- | --- |
|  | **N** | **Range** | **Range** | **Median** | **Range** | **Median** |
| **General CBT** |  |  |  |  |  |  |
| At least once | 13 |  |  |  | 35-98 | 93 |
| **Understanding and recognizing emotions** |  | 2-5 | 75-100 | 100 |  |  |
| At least once | 13 |  |  |  | 50-83 | 75 |
| At least twice | 13 |  |  |  | 8-58 | 25 |
| **Mindfulness essential** |  | 1-9 | 0-100 | 84.5 |  |  |
| At least once | 8 |  |  |  | 0-100 | 100 |
| At least twice | 8 |  |  |  | 0-100 | 50 |
| **Mindfulness optional** |  | 1-9 | 60-100 | 100 |  |  |
| At least once | 8 |  |  |  | 11-100 | 44 |
| At least twice | 8 |  |  |  | 0-88 | 13 |
| **Mini mindfulness essential** |  | 1-2 | 100-100 | 100 |  |  |
| At least once | 3 |  |  |  | 75-100 | 100 |
| At least twice | 3 |  |  |  | 0-75 | 0 |
| **Mini mindfulness optional** |  | 1-2 | 0-100 | 0 |  |  |
| At least once | 3 |  |  |  | 0-67 | 0 |
| At least twice | 3 |  |  |  | 0-0 | 0 |
| **CUES essential** |  | 1-5 | 60-100 | 90 |  |  |
| At least once | 6 |  |  |  | 14-86 | 50 |
| At least twice | 6 |  |  |  | 0-22 | 14 |
| **CUES optional** |  | 1-5 | 60-100 | 100 |  |  |
| At least once | 6 |  |  |  | 43-71 | 50 |
| At least twice | 6 |  |  |  | 0-50 | 23 |
| **Social anxiety essential** |  | 1-9 | 50-100 | 100 |  |  |
| At least once | 7 |  |  |  | 13-88 | 75 |
| At least twice | 7 |  |  |  | 0-75 | 25 |
| **Social anxiety optional** |  | 1-9 | 0-100 | 53.5 |  |  |
| At least once | 8 |  |  |  | 0-100 | 50 |
| At least twice | 8 |  |  |  | 0-67 | 13 |

*‘At least once’ and ‘At least twice’ refer to the number, range and median of essential interventions that were covered at least once or at least twice in the full course of a participant’s treatment.*

**Supplementary Table 4. Mean change rating in Target Situation from baseline to three months post-intervention**

| **Mean Target Situation vignette rating** | **Frequency by group**  N (% of vignettes rated) | | | |
| --- | --- | --- | --- | --- |
|  | **PAT-A© (N = 27)** | | **CCSP (N = 24)** | |
|  | **Impact on individual** | **Impact on others** | **Impact on individual** | **Impact on others** |
| Very much improved | 2 (7.4) | 0 (0.0) | 0 (0.0) | 0 (0.0) |
| Markedly improved | 0 (0.0) | 3 (11.1) | 3 (12.5) | 3 (12.5) |
| Definitely improved | 6 (22.2) | 1 (3.7) | 2 (8.3) | 0 (0.0) |
| Equivocally improved | 5 (18.5) | 3 (11.1) | 5 (20.8) | 1 (4.2) |
| No change | 10 (37.0) | 17 (63.0) | 10 (41.7) | 17 (70.8) |
| Equivocally worse | 2 (7.4) | 1 (3.7) | 4 (16.7) | 3 (12.5) |
| Definitely worse | 2 (7.4) | 2 (7.4) | 0 (0.0) | 0 (0.0) |
| Markedly worse | 0 (0.0) | 0 (0.0) | 0 (0.0) | 0 (0.0) |
| Disastrously worse | 0 (0.0) | 0 (0.0) | 0 (0.0) | 0 (0.0) |
| **Target Situation vignettes not available for rating** | **Frequency by group**  N (% of vignettes compared to baseline) | | | |
|  | **PAT-A**  **(N= 34)** | | **CCSP**  **(N=33)** | |
| Vignettes not rated due to the impact of COVID-19 | 5 (14.7) | | 3 (9.1) | |
| Vignettes missing at follow up | 2 (5.9) | | 6 (18.2) | |
| Total missing | 7 (20.6) | | 9 (27.3) | |

**Supplementary Table 5. Global Clinical Impression of Improvement (CGI-I) from baseline to three months post-intervention**

|  | **Frequency by group**  N (% of CGI-Is rated) | |
| --- | --- | --- |
| **Global Clinical Impression of Improvement (CGI-I) rating** | **PAT-A© (N = 13)** | **CCSP (N = 10)** |
| Very much improved | 0 (0.0) | 0 (0.0) |
| Much improved | 3 (23.1) | 3 (30.0) |
| Minimally improved | 5 (38.5) | 0 (0.0) |
| No change | 2 (15.4) | 3 (30.0) |
| Minimally worse | 3 (23.1) | 4 (40.0) |
| Much worse | 0 (0.0) | 0 (0.0) |
| Very much worse | 0 (0.0) | 0 (0.0) |
| **CGI-I not rated due to missing data** | **Frequency by group**  N (% of total participants in group) | |
|  | **PAT-A© (N = 17)** | **CCSP (N = 17)** |
| CGI-I not rated | 4 (23.5) | 7 (41.2) |

**Supplementary Table 6. Mean subscale and total scores for all outcome measures at baseline and follow-up**

| **Measure** | **PAT-A**  Mean (SD) | | | **CCSP**  Mean (SD) | | |
| --- | --- | --- | --- | --- | --- | --- |
|  | **Baseline** | **Immediate** | **3 months** | **Baseline** | **Immediate** | **3 months** |
| **ASA-A**  *Higher scores indicate greater anxiety.* |  |  |  |  |  |  |
| *N* | 17 | 12 | 13 | 16 | 11 | 10 |
| *Social anxiety (max= 18)* | 13.3 (3.9) | 12.1 (3.4) | 13.5 (4.3) | 13.6 (3.5) | 12.1 (3.4) | 12.5 (3.9) |
| *Anxious arousal (max = 27)* | 13.8 (5.4) | 11.3 (6.2) | 14.5 (6.9) | 11.3 (4.5) | 9.9 (2.9) | 11.0 (4.3) |
| *Uncertainty (max = 15)* | 12.0 (3.0) | 8.9 (4.0) | 10.9 (3.2) | 10.9 (2.9) | 10.5 (3.4) | 10.1 (3.3) |
| *Total (max = 60) (α = .91)* | 39.2 (10.9) | 32.3 (11.9) | 39.0 (13.4) | 35.5 (9.2) | 32.5 (9.1) | 33.9 (11.1) |
| **HADS**  *Higher scores indicate greater difficulties.* |  |  |  |  |  |  |
| *N* | 17 | 12 | 13 | 15 | 11 | 10 |
| *Anxiety (α = .71) (max = 21)* | 15.3 (3.7) | 12.6 (5.1) | 14.3 (4.6) | 13.4 (3.3) | 12.6 (3.3) | 12.1 (3.7) |
| *Depression (α = .72)  (max = 21)* | 11.7 (4.4) | 10.6 (4.4) | 11.8 (5.4) | 9.2 (3.6) | 8.7 (3.6) | 9.8 (4.0) |
| **WHOQoL-BREF**  *Range= 0-100 where 0 is the worst QoL and 100 is the best*  *(α = .91)* |  |  |  |  |  |  |
| *n* | 17 | . | 13 | 15 | . | 9 |
| *Physical health* | 42.0 (23.9) | . | 38.2 (19.3) | 45.6 (17.1) | . | 41.6 (20.4) |
| *Psychological* | 30.9 (17.7) | . | 21.6 (16.8) | 30.9 (21.7) | . | 30.5 (8.5) |
| *Social relationships* | 38.9 (24.5) | . | 39.5 (25.3) | 39.8 (22.5) | . | 36.0 (28.7) |
| *Environment* | 53.1 (18.0) | . | 51.2 (21.2) | 56.4 (15.9) | . | 51.9 (14.6) |
| **Disabilities Module**  *Range= 0-100 where 0 is the worst QoL and 100 is the best* |  |  |  |  |  |  |
| *n* | 17 | . | 13 | 15 | . | 9 |
| *Discrimination* | 38.1 (25.6) | . | 33.2 (25.1) | 42.6 (18.8) | . | 41.5 (15.4) |
| *Autonomy* | 46.4 (24.0) | . | 40.2 (22.2) | 50.9 (19.0) | . | 46.7 (18.3) |
| *Inclusion* | 26.4 (16.8) | . | 24.2 (19.6) | 30.0 (19.2) | . | 27.3 (18.9) |
| *Total (α = .86)* | 34.3 (17.7) | . | 30.5 (17.5) | 38.3 (15.1) | . | 35.7 (12.2) |
| **ASQoL**  *Range= 0-5 where 0 is the worst QoL and 5 is the best* |  |  |  |  |  |  |
| *n* | 16 | . | 13 | 16 | . | 9 |
| *Total (α = .79)* | 2.7 (.7) | . | 2.8 (.7) | 2.7 (.8) | . | 2.7 (.7) |
| **EQ-5D-5L**  *Range= 0-5 where higher scores indicate greater difficulties* |  |  |  |  |  |  |
| *n* | 16 | . | 13 | 16 | . | 10 |
| *Mobility* | 1.6 (1.0) | . | 2.0 (1.2) | 1.4 (.6) | . | 1.6 (1.1) |
| *Self-care* | 1.7 (.9) | . | 2.2 (1.1) | 1.8 (1.0) | . | 1.7 (1.1) |
| *Usual activities* | 2.8 (1.1) | . | 2.9 (1.0) | 2.6 (1.1) | . | 2.5 (.9) |
| *Pain/ discomfort* | 2.1 (1.4) | . | 2.6 (1.0) | 1.8 (.9) | . | 2.2 (.9) |
| *Anxiety/ depression* | 3.6 (.8) | . | 3.9 (.8) | 3.8 (1.1) | . | 3.7 (.9) |
| *Current health*  *Range= 0-100 where 0 is the worst health and 100 is the best* | 48.5 (22.2) | . | 44.5 (20.7) | 50.4 (17.2) | . | 53.5 (22.1) |

α = Cronbach’s alpha
